# Dr. Dumont

**Published:** 1852-04

**Authors:** 


***Dr. Dumont.***—The many friends of Dr. S. H. Dumont, who graduated with so much honor at the Baltimore College of Dental Surgery, in 1850, will be glad to learn that he is gradually but surely introducing, in Brussels, the more efficient and serviceable operations of our country.

Dr. Dumont has much prejudice and ill-will to combat, but is determined to succeed:—and from what we know of his skill and integrity, we predict for him a brilliant professional career when the Belgians have had some further opportunities of comparing his operations, with those generally performed on the continent of Europe. We wish him success for his own good; but the consequent good to his patients will be a myriad-fold greater.

